# Arsenic and Erectile Dysfunction: Drinking Contaminated Well Water Increases Risk

**DOI:** 10.1289/ehp.116-a172b

**Published:** 2008-04

**Authors:** Kris Freeman

Age is the most common risk factor for erectile dysfunction (ED), the consistent or recurrent inability to attain and/or maintain a penile erection sufficient for sexual performance. The correlation between age and ED is attributed to declines in testosterone levels; growing evidence links the condition to cardiovascular disease (CVD) as well. Now researchers from Taiwan have found a direct correlation between ED, the decline of testosterone, and exposure to arsenic via well water—a connection of potential concern for the millions of men worldwide who drink groundwater contaminated with naturally occurring arsenic **[*EHP* 116:532–536; Hsieh et al.]**.

Besides its association with ED, CVD has also been linked to chronic arsenic exposure, perhaps by reducing the synthesis of nitric oxide (NO), which is involved in the control of smooth muscle in blood vessels. In the penis, NO activates cyclic guanosine monophosphate, which dilates blood vessels, allowing the penis to become engorged with blood. Testosterone can regulate activity of the enzyme nitric oxide synthase, which creates NO.

The researchers measured free testosterone levels in the blood of 129 men with ED and 48 without. The average age of the study participants was about 67 years. Sixty-six of the participants were from an arsenic-endemic area in northeast Taiwan where residents have used contaminated artesian well water for more than 50 years. Arsenic exposure was determined by analysis of participants’ well water.

As arsenic exposure of participants increased, so did the risk of ED. The prevalence of ED was 83.3% among men from the arsenic-endemic area compared with 66.7% among men outside this area. Moreover, as the arsenic exposure of the participants increased, their testosterone levels decreased.

The risk of carotid atherosclerosis increased with increasing levels of exposure, but only in men who drank well water containing arsenic concentrations higher than 50 ppb. These men also had a significantly higher risk of ED than men who drank water with arsenic concentrations below 50 ppb, even after adjustments for testosterone levels. Other risk factors for ED did not affect the associations.

According to the authors, arsenic exposure appears to increase ED risk by decreasing testosterone levels. However, they speculate that other factors are at work, as decreases in testosterone did not account for all the ED found in men with high arsenic exposures. The presence of oxygen free radicals can inhibit the synthesis of NO and impair blood vessel function. Therefore, the researchers suggest that oxidative stress from high arsenic exposure may also increase ED risk.

